# Ginseng Protects Against Respiratory Syncytial Virus by Modulating Multiple Immune Cells and Inhibiting Viral Replication

**DOI:** 10.3390/nu7021021

**Published:** 2015-02-04

**Authors:** Jong Seok Lee, Yu-Na Lee, Young-Tae Lee, Hye Suk Hwang, Ki-Hye Kim, Eun-Ju Ko, Min-Chul Kim, Sang-Moo Kang

**Affiliations:** 1 Center for Inflammation, Immunity & Infection, Institute for Biomedical Sciences, Georgia State University, Atlanta, GA 30303, USA; E-Mails: jongseoklee78@gmail.com (J.S.L.); mistybluerain7@gmail.com (Y.-N.L.); leechard75@gmail.com (Y.-T.L.); hshwang33@gmail.com (H.S.H.); kihyekim4282@gmail.com (K.-H.K.); ej.ko226@gmail.com (E.-J.K); mckim001@gmail.com (M.-C.K.); 2 National Institute of Biological Resources, Incheon 404–708, Korea; 3 Animal and Plant Quarantine Agency, 175 Anyangro, Anyangsi, Gyeonggido 430–757, Korea

**Keywords:** ginseng, respiratory syncytial virus, antiviral activity, cytokines, Immuno-modulatory effects

## Abstract

Ginseng has been used in humans for thousands of years but its effects on viral infection have not been well understood. We investigated the effects of red ginseng extract (RGE) on respiratory syncytial virus (RSV) infection using *in vitro* cell culture and *in vivo* mouse models. RGE partially protected human epithelial (HEp2) cells from RSV-induced cell death and viral replication. In addition, RGE significantly inhibited the production of RSV-induced pro-inflammatory cytokine (TNF-α) in murine dendritic and macrophage-like cells. More importantly, RGE intranasal pre-treatment prevented loss of mouse body weight after RSV infection. RGE treatment improved lung viral clearance and enhanced the production of interferon (IFN-γ) in bronchoalveolar lavage cells upon RSV infection of mice. Analysis of cellular phenotypes in bronchoalveolar lavage fluids showed that RGE treatment increased the populations of CD8^+^ T cells and CD11c^+^ dendritic cells upon RSV infection of mice. Taken together, these results provide evidence that ginseng has protective effects against RSV infection through multiple mechanisms, which include improving cell survival, partial inhibition of viral replication and modulation of cytokine production and types of immune cells migrating into the lung.

## 1. Introduction

Respiratory syncytial virus (RSV), a member of the *Paramyxoviridae* family, is a negative single-stranded RNA virus. RSV is the leading cause of serious respiratory disease in infants and immune-suppressed populations [1,2]. The mechanisms of causing disease by respiratory viruses are not fully understood. During the primary RSV infection in the respiratory tracts, lung epithelium and alveolar macrophages are likely to be the major cell types infected, which subsequently trigger the production of a wide range of T helper type 1 and type 2 cytokines and chemokines [3]. Recruitment of inflammatory cells into the lung plays a central role in determining a disease outcome during RSV infection [4,5,6]. RSV infection is known to cause enhanced expression of cytokines such as interleukin (IL)-6, IL-10, and tumor necrosis factor (TNF)-α, and the chemokine such as IL-8, interferon (IFN)-inducible protein (IP)-10, growth-regulated protein (GRO), and RANTES in different cell types by *in vitro* culture studies [7,8]. In clinical studies, it was reported that high levels of proinflammatory cytokines including IL-4, IL-5 and IL-6 were associated with acute bronchiolitis in RSV-infected children [3]. These data were consistent with excessive T helper type 2 and/or deficient type 1 immune responses in RSV bronchiolitis [1,9]. Both innate and adaptive immune responses are thought to contribute to the development of bronchiolitis in RSV infection [10]. Dendritic cells are uniquely positioned to link innate to adaptive immune responses and may therefore play a role in modulating bronchiolitis [11].

Herbal medicines have been used for thousands of years, and thus hold a great promise for their usefulness in treating medical illnesses or in improving physical performance. Among many herbal medicines, *Panax ginseng C. A. Meyer* mainly produced in Korea, China, and America, is one of the most commonly used ginseng plants [12,13]. Ginseng has been shown to display immunomodulatory effects either in an immuno-stimulatory or in an immuno-suppressive manner depending on disease environment [14]. It was reported that ginseng could stimulate different immune cells, indicating its immuno-stimulatory function [15,16]. In other studies, a polysaccharide component of ginseng was shown to suppress early acute inflammatory responses, contributing to the protection of mice from *Staphylococcus aureus*-induced sepsis, supporting a role of ginseng as an anti-inflammatory function [17,18]. In addition, ginseng is known to have beneficial pharmacological effects on hypodynamia, anorexia, shortness of breath, palpitations, insomnia, impotence, hemorrhage, and diabetes [19]. However, the potential effects of ginseng on RSV infection remain unknown.

In the present study, we investigated the potential effects of red ginseng extract (RGE) on RSV-induced cytopathogenic formation and viral replication in human epithelial cells. We examined whether RGE could inhibit RSV-induced pro-inflammatory cytokines in murine dendritic and macrophage-like cell lines. In addition, we evaluated the potential immunomodulatory functions of RGE after RSV viral infection in a mouse model.

## 2. Materials and Methods

### 2.1. Cells, Virus, Reagents

The RSV A2 strain was originally provided by Dr. Barney Graham (NIH, Bethesda, MD, USA) as previously described [20,21]. HEp2 cells were obtained from American Type Culture Collection and macrophage-like cell line RAW264.7 was a gift from Dr. Martin J. D’Souza (College of Pharmacology and Health Sciences, Mercer University, Atlanta, GA, USA). Dendritic cell line DC2.4 was also kindly provided by Dr. Martin J. D’Souza, and cultured and used as previously described [22,23]. Korean red ginseng extract (RGE), a concentrated form of the commercial ginseng product was obtained from the Korea Ginseng Corporation (Daejeon, Korea). Briefly, fresh roots of the *Panax ginseng* that had grown for six years were washed, steamed at 100 °C for 2 to 3 h and dried. The dried red ginseng roots after the steaming process were boiled in 4 to 5 volumes of water for 3 h and the supernatants (600 g, 30 min) were concentrated. This preparation obtained after centrifugation was designated “red ginseng extract (RGE)” (approximately 36% water content) which contains approximately 1.8% to 2.3% ginsenosides (18–23 mg ginsenosides/g red ginseng extract powder). Polyclonal goat anti-RSV antibody and mouse anti-RSV fusion protein were purchased from Millipore (Billerica, MA, USA). Secondary HRP-conjugated anti-mouse antibody was purchased from Southern Biotech (Birmingham, AL, USA). Fetal bovine serum (FBS), penicillin-streptomycin, RPMI1640, and Dulbecco’s modified Eagle’s medium (DMEM) were purchased from GIBCO (Grand Island, NY, USA). All other chemicals were analytical grade.

### 2.2. Preparation of RSV Stock

HEp2 cells were grown in tissue culture flasks in DMEM containing 10% FBS. RSV was added, and virus adsorption was carried out in medium without serum for 1 h at 37 °C with 5% CO_2_. DMEM with 5% FBS was added to the flask and incubated for 3–5 days. RSV-infected cells were collected using a cell scraper, sonicated and centrifuged at 2000 rpm for 10 min at 4 °C, and the supernatants were titrated by an immunoplaque assay as described [20,21] and stored at −80 °C.

### 2.3. RSV Immunoplaque Assay

HEp2 cells were grown in 12-well plates until confluent. Virus stock or lung homogenates from infected mice were serially diluted in DMEM media without FBS. Virus samples were added to the plates and incubated for 1 h at 37 °C. Each well received 1 mL of overlay and was incubated 3–6 days at 37 °C. Cells were fixed with ice-cold acetone-methanol (60:40) for 10 min. After air-drying, anti-F monoclonal antibody and then HRP-conjugated anti-mouse IgG antibodies were used. Individual plaques were developed using DAB substrate (Invitrogen, Camarillo, CA, USA).

### 2.4. Cell Viability Assay

The effect of RGE and RSV A2 virus on the cell viability was determined using the (3-(4,5-dimethylthiazol-2-yl)-2,5-diphenyltetrazolium) bromide (MTT) assay, which is based on the reduction of a tetrazolium salt by mitochondrial dehydrogenase in viable cells [24]. After various treatments, 50 μL of the MTT stock solution (2 mg/mL) was then added to each well to attain a total reaction volume of 200 μL. After incubation for 2 h at 37 °C, the formazan crystals in each well were dissolved in isopropyl alcohol, and the absorbance was determined at 570 nm. Cell viability was expressed as a percentage with the control cells treated with vehicle as 100%.

### 2.5. Cytopathogenic Effect (CPE) Reduction Assay

The cytopathogenic effect (CPE) was determined as previously described [25]. Confluent cell monolayers grown in 96-well plates were infected with RSV at the indicated multiplicities of infection (MOIs). The virus-induced CPE was recorded at 48 h post infection.

### 2.6. Assays for Cytokines

After various treatments, culture supernatants were collected from dendritic cells and macrophages treated with or without RSV and RGE. Concentrations of TNF-α in the culture supernatants were determined using ELISA kit (eBioscience, San Diego, CA, USA) according to the manufacturer’s instructions.

### 2.7. Treatment of Mice with RGE and RSV A2 Virus

RGE was dissolved in sterile PBS and filtered through 0.45 μm Millipore membrane. For animal experiments, 6–8 weeks old female BALB/c mice (Harlan Laboratories, Indianapolis, IN, USA) were used. To determine the preventative effects of RGE treatment on RSV infection, BALB/c mice (*n* = 5 per group) were pretreated one time intranasally with RGE (4 mg per mouse) 1 day prior to infection with RSV. To determine the protective effects of RGE treatment on RSV infection, BALB/c mice (*n* = 5 per group) were infected intranasally with a mixture of RGE (4 mg per mouse) and RSV. As a control, BALB/c mice (*n* = 5 per group) were intranasally infected with RSV (1 × 10^5^ PFU or 1 × 10^6^ PFU). Mice were anesthetized by isoflurane inhalation before treatment with RGE or RSV. Mice were monitored daily to record weight changes. Full details of this study and all animal experiments presented in this manuscript were approved by the Georgia State University (GSU) Institutional Animal Care and Use Committee (IACUC) review board on 31 October 2013 (A11026) and conducted under the guidelines of the IACUC. GSU IACUC operates under the federal Animal Welfare Law (administered by the USDA) and regulations of the Department of Health and Human Services.

### 2.8. Lung Virus Titer and Cytokine Assays

Mice were anesthetized with isoflurane and exsanguinated after severing of the right caudal artery. The individual lungs were removed aseptically at day 5 post challenge, and lung extracts were prepared as homogenates after challenge using frosted glass slide [21]. The homogenates were centrifuged at 2000 rpm for 10 min to collect supernatants. The virus titer in the supernatants was determined by an immunoplaque assay. Cytokine ELISA was performed as described previously [26]. Ready-Set-Go IFN-γ kits (eBioscience, San Diego, CA, USA) were used for detecting cytokine levels in bronchoalveolar lavage (BAL) fluids following the manufacturer’s recommended procedures [27].

### 2.9. Preparation of Bronchoalveolar Lavage (BAL) and Flow Cytometric Analysis

Five days after RSV infection, mice were sacrificed to collect BAL fluids (BALF) and lung samples. BALF samples were obtained by infusing 1 mL of PBS into the lungs via the trachea using a 25-gauge catheter (Exelint International Co., Los Angeles, CA, USA) as described [27]. Cells from BALF were stimulated with RSV F peptide (1 μg/mL) or RSV G peptide (1 μg/mL) for 4 h. After staining with surface antibodies (anti-CD45, CD3, CD8α, F4/80, CD11b, CD11c antibodies from eBiosciences), intracellular IFN-γ cytokine staining was followed by manufacturer’s manuals (BD Cytofix/Cytoperm™ Fixation/Permeabilization Solution Kit). The percentage of gated cells was calculated by Flow Jo software (Tree Star Inc., San Carlos, CA, USA).

### 2.10. Statistical Analysis

Data were expressed as means ± standard error (SEM), and the results were taken from at least three independent experiments performed in triplicate. The data were analyzed by Student’s *t*-test to evaluate significant differences. A level of *p* < 0.05 was regarded as statistically significant.

## 3. Results

### 3.1. Influence of RGE on RSV Replication in Human Epithelial Cells

**Figure 1:**
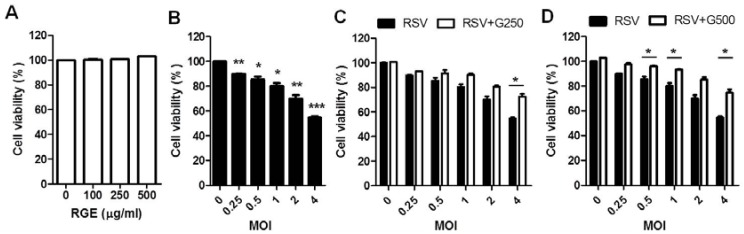
Effects of red ginseng extract (RGE) on respiratory syncytial virus (RSV)-induced cytopathogenic effects. (**A**) Effects of RGE concentrations on the growth of HEp2 cells; (**B**) Effects of different RSV MOIs on the viability of HEp2 cells. * *p* < 0.05; ** *p* < 0.01; *** *p* < 0.001 *vs.* non-infected cells; (**C**,**D**) Effects of different RGE concentrations on the viability of HEp2 cells infected with RSV at different MOIs 48 h post infection. HEp2 cells were continuously treated with RGE starting 24 h prior to infection and during the infection period. Values are the mean ± SEM. * *p* < 0.05. G250: 250 μg RGE per mL, G500: 500 μg RGE per mL.

**Figure 2:**
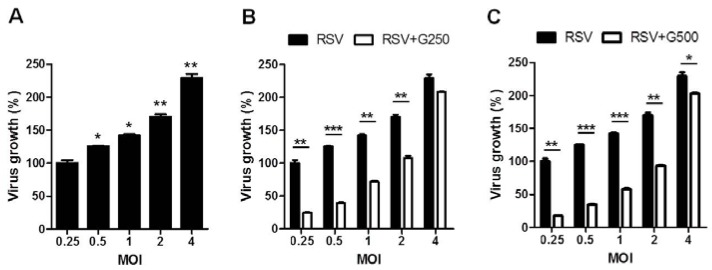
Red ginseng extract (RGE) inhibits respiratory syncytial virus (RSV) replication in human epithelial cells. (**A**) Growth of RSV in human epithelial cell line HEp2 cells. Values are the mean ± SEM. * *p* < 0.05; ** *p* < 0.01 *vs.* RSV-infected control at a MOI of 0.25; (**B**,**C**) Effects of different RGE concentrations on the growth of RSV in HEp2 cells infected with RSV at different MOIs 48 h post infection. Values are presented as mean ± SEM of three independent experiments and are expressed as the percentage of virus growth relative to the value of RSV infection at a MOI of 0.25. * *p* < 0.05; ** *p* < 0.01; *** *p* < 0.001. G250: 250 μg RGE per mL, G500: 500 μg RGE per mL.

Since epithelial cells are the primary targets of RSV infection [28], we investigated the possible effects of RGE on RSV infection in human epithelial (HEp2) cells. Confluent HEp2 cell layers were infected with RSV at different MOIs in the presence or absence RGE treatment. If not otherwise stated, RGE was continuously present in cell culture media starting with a day pre-infection period and during the infection period of two days. RGE at the concentration of 500 μg/mL did not affect HEp2 cell viability (Figure 1A). Infection with higher MOIs of RSV induced more cell death (Figure 1B). RGE treatment of human epithelial HEp2 cells during RSV infection partially prevented RSV-induced cell death (Figure 1C,D). More significantly, treatment with RGE reduced the production of RSV infectious viral titers (Figure 2). In particular, the effects of RGE on lowering RSV replication in HEp2 cells were more pronounced at lower MOIs of RSV (Figure 2).

### 3.2. Effects of RGE on RSV-Induced Cytokine Production in Dendritic and Macrophage Cells

**Figure 3:**
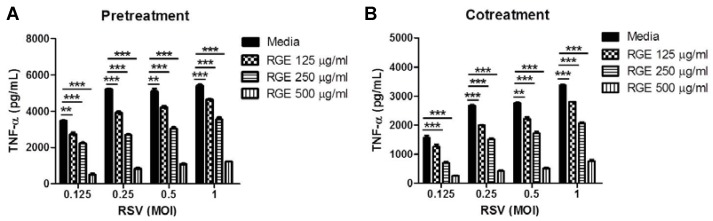
Red ginseng extract (RGE) inhibits respiratory syncytial virus (RSV)-induced inflammatory cytokine production in macrophages. RAW264.7 macrophages were mock-infected or infected with RSV at different MOIs. RAW264.7 macrophages were treated with RGE 24 h prior to infection or during the infection period. Concentrations of TNF-α in the culture supernatants were determined using ELISA kit. (**A**) Pre-treatment of RGE on RAW264.7 macrophages infected with RSV at different MOIs; (**B**) Co-treatment of RGE on RAW264.7 macrophages infected with RSV at different MOIs. Values are the mean ± SEM. * *p* < 0.05; ** *p* < 0.01; *** *p* < 0.001.

**Figure 4:**
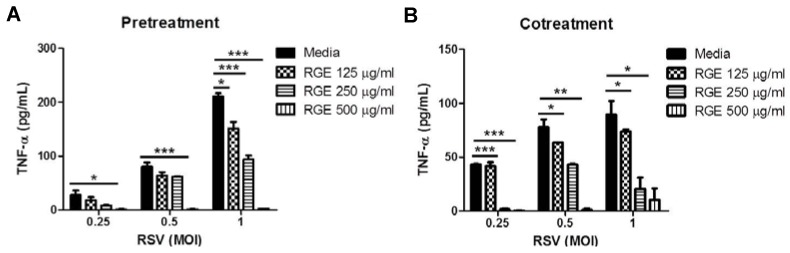
Red ginseng extract (RGE) inhibits respiratory syncytial virus (RSV)-induced cytokine production in dendritic cells. DC2.4 dendritic cells were mock-infected or infected with RSV at different MOIs. DC2.4 dendritic cells were treated with RGE 24 h prior to infection or during the infection period. Concentrations of TNF-α in the culture supernatants were determined using an ELISA kit. (**A**) Pre-treatment of RGE on DC2.4 dendritic cells infected with RSV at different MOIs; (**B**) Co-treatment of RGE on DC2.4 dendritic cells infected with RSV at different MOIs. Values are the mean ± SEM. * *p* < 0.05; ** *p* < 0.01; *** *p* < 0.001.

RSV infection can cause inflammatory pneumonia in lungs. To investigate whether RGE could inhibit RSV-induced pro-inflammatory cytokine production, RAW264.7 cells, a macrophage cell line, were infected with different MOIs of RSV in the presence or absence of RGE at various concentrations (Figure 3). It has been shown that RSV increased TNF-α expression by macrophages after *in vitro* infection [29]. In line with this, RSV-infected RAW264.7 cells significantly induced the production of a pro-inflammatory cytokine TNF-α compared to mock-treated cells (Figure 3). RGE treatment significantly inhibited the production of pro-inflammatory cytokine TNF-α induced by RSV infection in the RAW264.7 macrophage cell line (Figure 3). To further test whether RGE treatment would inhibit the production of pro-inflammatory cytokines induced by RSV infection, DC2.4 cells, a dendritic cell line, were infected with different MOIs of RSV in the presence or absence of various concentrations of RGE. The production of cytokine TNF-α in DC2.4 cells was more responsive to the different MOIs of RSV infection than the TNF-α production in RAW264.7 cells (Figure 4). A similar pattern of RGE dose-dependent inhibition in the TNF-α production was observed in the RSV-infected DC2.4 dendritic cell line (Figure 4). Therefore, these results indicate that RGE could inhibit the production of pro-inflammatory cytokine TNF-α induced by RSV infection in a dose-dependent manner.

### 3.3. RGE Treatment Improves Clinical Outcomes upon RSV Infection of Mice

**Figure 5:**
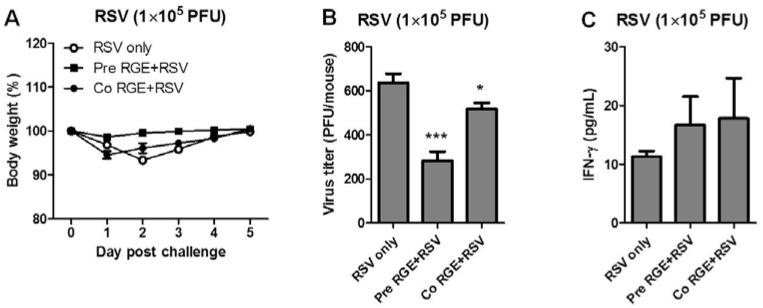

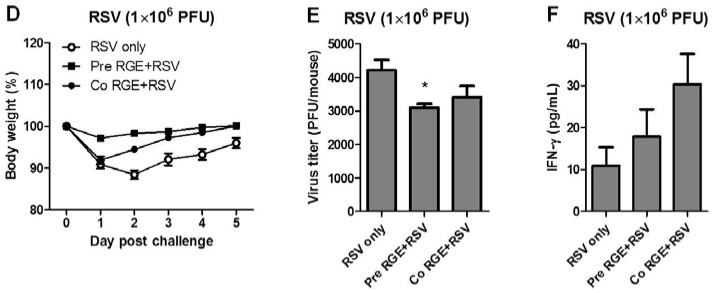
Pretreatment with red ginseng extract (RGE) improves clinical outcomes in mice upon infection with respiratory syncytial virus (RSV). Changes in body weight (**A**,**D**), lung viral titers (**B**,**E**), and bronchoalveolar lavage fluids (BALF) cytokine level of interferon (IFN)-γ (**C**,**F**) were determined at day 5 post RSV infection. Values are the mean ± SEM. * *p* < 0.05; ** *p* <0.01; *** *p* < 0.001 *vs.* RSV-infected control. RSV only: naïve mice infected with RSV in PBS, Pre RGE + RSV: naïve mice were pretreated intranasally once with RGE (4 mg per mouse) 24 h prior to infection with RSV, Co RGE + RSV: naive mice were treated once intranasally with a mixture of RGE (4 mg per mouse) and RSV.

An *in vivo* animal model would provide more relevant information on RGE effects on RSV infection. To examine whether RGE could confer protection against RSV infection, BALB/c mice were intranasally pretreated once with RGE (4 mg per mouse) 1 day prior to infection with RSV (Pre RGE + RSV) or intranasally infected with RSV (1 × 10^5^ or 1 × 10^6^ PFU/mouse) as a mixture with RGE (Co RGE + RSV) (Figure 5). Body weight changes were daily monitored as an indicator for morbidity (Figure 5A). Without RGE treatment, pronounced loss in body weight of infected naïve mice were observed in an RSV infection dose dependent manner. A high dose of RSV (1 × 10^6^ PFU/mouse) caused more weight loss than a low dose RSV (1 × 10^5^ PFU/mouse) (Figure 5A,D). Pretreatment of mice with RGE prior to infection prevented weight loss in mice subsequently infected with RSV compared to untreated RSV infected mice (1 × 10^5^ PFU per mouse) (Figure 5A,D). RGE effects on preventing weight loss were more prominent when a high dose of RSV (1 × 10^6^ PFU per mouse) was used (Figure 5A,D). Co-treatment of mice with RGE at the time of RSV infection also showed some effects on preventing severe weight loss compared to the control group without RGE (Figure 5D) but the degree of preventing weight loss was lower than that by RGE pretreatment. To better understand protective parameters by RGE treatment, we determined viral loads in lungs and IFN-γ cytokine in bronchoalveolar lavage fluids (BALF) at day 5 post infection (Figure 5). RGE-pretreated mice exhibited significantly lower lung viral titers compared to those of untreated RSV infected mice (Figure 5B,E). In addition, RGE-treated mice showed a trend of increasing the levels of IFN-γ cytokine in BALF although there were no statistically significant differences compared to untreated RSV-infected control mice (Figure 5C,F). Pretreatment with RGE showed more protective effects on preventing weight loss and controlling lung viral loads than treatment with RGE at the time of RSV infection. Overall, RGE treatment improved the clinical outcomes of mice upon RSV infection.

### 3.4. RGE Modulates Bronchoalveolar Immune Cells upon RSV Infection of Mice

**Figure 6:**
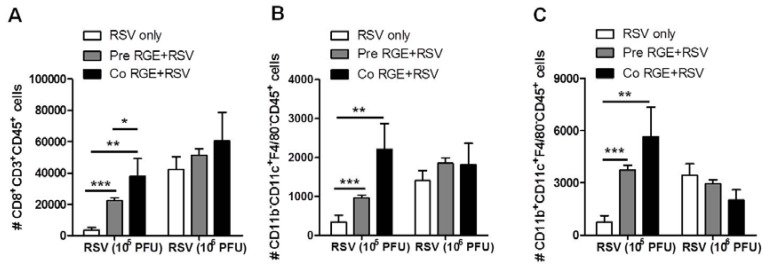
*In vivo* effects of red ginseng extract (RGE) on phenotypes of bronchoalveolar lavage cells from mice infected with respiratory syncytial virus (RSV). Bronchoalveolar lavage (BAL) was collected five days after RSV challenge, stained with CD45, CD3, CD8α, CD11b, CD11c, and analyzed by flow cytometry. (**A**) CD8^+^CD3^+^CD45^+^ T cells; (**B**) CD11b^−^CD11c^+^F4/80^−^CD45^+^ dendritic cells; (**C**) CD11b^+^CD11c^+^F4/80^−^CD45^+^ dendritic cells. Values are the mean ± SEM. * *p* < 0.05; ** *p* < 0.01; *** *p* < 0.001. RSV only: naïve mice infected with RSV in PBS, Pre RGE + RSV: naïve mice were pretreated intranasally once with RGE (4 mg per mouse) 24 h prior to infection with RSV, Co RGE + RSV: naive mice were once cotreated intranasally with the mixture of RGE (4 mg per mouse) and RSV.

**Figure 7:**
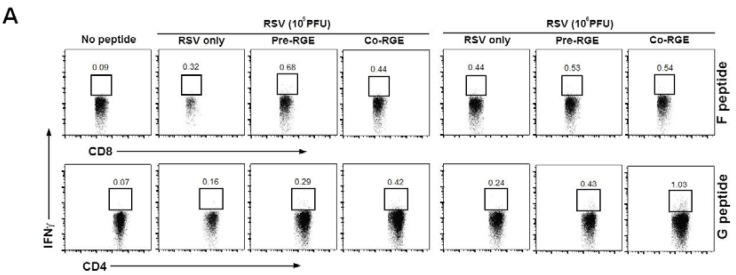

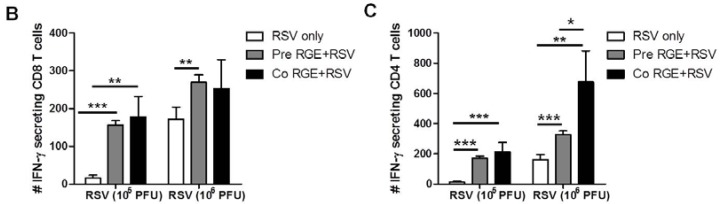
Phenotypes of interferon (IFN)-γ secreting T cells in bronchoalveolar lavage fluids (BALF) from mice with RGE treatment and respiratory syncytial virus (RSV) infection. BAL cells were harvested, stained with CD45, CD3, CD8α, CD11b, CD11c, and IFN-γ antibodies, and analyzed by flow cytometry. IFN-γ secreting T cells were presented as total numbers per mouse. (**A**) Flow cytometry of RSV F peptide specific CD8^+^IFN-γ^+^ T cells and RSV G peptide specific CD8^+^IFN-γ^+^ T cells. Numbers in dot plots indicate the cell percentages of double positive population; (**B**) Numbers of RSV F peptide specific CD8^+^IFN-γ^+^ T cells; (**C**) Numbers of RSV G peptide specific CD8^+^IFN-γ^+^ T cells. Values are the mean ± SEM. * *p* < 0.05; ** *p* < 0.01; *** *p* < 0.001. RSV only: naïve mice infected with RSV in PBS, Pre red ginseng extract (RGE) + RSV: naïve mice were pretreated once intranasally with RGE (4 mg per mouse) 24 h prior to infection with RSV, Co RGE + RSV: naive mice were treated once intranasally with the mixture of RGE (4 mg per mouse) and RSV.

As lung is a major organ of RSV disease, we determined the phenotypes of immune cells in BAL cells from RGE-pretreated or co-treated mice upon RSV infection (Figure 6). Flow cytometry analysis showed that the numbers of CD8^+^CD3^+^CD45^+^ T cells in mice pretreated and cotreated with RGE were significantly increased compared to those in untreated mice with RSV infection (Figure 6A). Dendritic cells play an important role in T cell differentiation and in the initiation of protective immunity or pathogenic responses to pathogens. In mice, all dendritic cells express the integrin CD11c and their subsets are further defined based on the expression of the myeloid marker CD11b [30]. The numbers of CD11b^−^CD11c^+^F4/80^−^CD45^+^ and CD11b^+^CD11c^+^F4/80^−^CD45^+^ phenotypic dendritic cells in BAL from mice pretreated and cotreated with RGE were increased compared to those in untreated mice that were infected with a low dose of RSV (1 × 10^5^ PFU) (Figure 6B,C). At a high dose of RSV (1 × 10^6^ PFU) infection of mice, the cellularity of both CD8^+^ T cells and CD11c^+^ dendritic cells was significantly increased (Figure 6). In contrast to the low dose RSV groups, treatment with RGE did not significantly change the cellularity of these BAL cells in groups of mice that were infected with a high dose of RSV (1 × 10^6^ PFU). These results suggest that RGE treatment can increase dendritic cell populations to a certain level depending on a dose of RSV infection. To further understand the effects of RGE in conferring protection against RSV infection, the levels of intracellular cytokine IFN-γ secreting cells in BAL from mice were determined by flow cytometry at day 5 post infection. When we determined the levels of intracellular IFN-γ secreting BAL cells using flow cytometry, the numbers of RSV F-specific IFN-γ-secreting CD8^+^ T cells in the mice pretreated and co-treated with RGE were significantly increased compared to those in the untreated mice with RSV infection (Figure 7A,B). In addition, we observed that the numbers of RSV-specific IFN-γ secreting CD4^+^ and CD8^+^ T cells in mice pretreated and cotreated with RGE was significantly enhanced compared to those in the RSV-infected untreated mice (Figure 7A,C). The effects of RGE treatment on increasing IFN-γ secreting CD4^+^ and CD8^+^ T cells in BALF were more prominent when a low dose of RSV (1 × 10^5^ PFU) was used to infect mice. Therefore, the possible mechanism is that RGE treatment exhibits protective effects on RSV infection via modulation of IFN-γ-secreting T cell responses.

## 4. Discussion

Viral respiratory tract infections can lead to severe disease at all ages, but prevention is not available for most respiratory viruses. Thus, effective disease-limiting therapy is urgently required. *Panax ginseng* is one of the most well studied herbal medicines and appears to have multiple effects including an immunomodulatory function and a potential antiviral activity. However, the potential immunomodulatory and antiviral effects of ginseng on RSV infection remain unknown. We have investigated whether RGE has preventative and protective effects against RSV infection. RGE protected human epithelial cells from RSV-induced cytopathogenic formation. RGE treatment significantly inhibited the replication of RSV in human epithelial cells *in vitro*. In addition, RGE interfered with RSV-induced pro-inflammatory cytokine production in murine dendritic and macrophage-like cell lines. More importantly, RGE pre-treatment or co-treatment of mice exhibited beneficial effects on diminishing illness as demonstrated by reduced weight loss during RSV infection in a mouse model. The treatment of RGE improved lung viral clearance and enhanced the production of IFN-γ cytokine secreting cells. Overall, these results suggest that ginseng might have preventative and protective effects against RSV infection. RSV was found to induce significant cell death of HEp2 cells, which probably due to RSV-induced damage to the cells as previously reported [3,31]. The treatment of RGE on RSV-infected epithelial cells resulted in partial protection from cell death induced by RSV infection and also significant inhibition of the *in vitro* growth of RSV. Inhibition of RSV-induced cell death by RGE treatment might have been associated with antioxidant activity of RGE, at least in part through interference with RSV-induced cellular oxidative damage [32,33,34,35]. At low MOIs of 0.25 to 0.5, RSV-induced cell death was minimal but the RGE-mediated inhibition of RSV growth was significantly higher than that observed at the high dose of MOI. This suggests that RGE may have an antiviral function on RSV, which is more effective at low MOIs. In contrast to the effect of RGE on RSV growth, the RGE-mediated inhibition of TNF-α inflammatory cytokine production was more dependent on the concentration of RGE rather than RSV MOI (Figure 3). It seems to be possible that RGE may have dual mechanisms of anti-RSV activity and an anti-inflammatory effect upon RSV infection. In general, control of lung viral loads is an important parameter in assessing protection against RSV since a positive correlation was reported between viral replication and clinical disease during natural or experimental infections [36,37]. In an *in vivo* model, the treatment of mice with RGE resulted in lowering lung viral loads upon RSV infection. A possible mechanism is that RGE-mediated inhibition of both RSV replication and RSV-induced cell death might have contributed to improving lung viral clearance in RGE-treated mice as well as lowering or preventing weight loss. The severity of human respiratory virus disease has been associated with hypercytokinemia. A wide range of pro-inflammatory cytokines and chemokines such as TNF-α and IL-8 are produced by airway epithelial cells and macrophages in response to viral infection, leading to recruitment and activation of macrophages, dendritic cells and neutrophils, all of which are involved in viral lung inflammation [38,39,40]. T helper type 2 immune responses such as high levels of IL-4 *versus* IFN-γ and the release of IL-5 and IL-13 cytokines are known to contribute to eosinophil recruitment to the lung, goblet cell formation from epithelial cells, mucus production and airway hyperresponsiveness [41]. The broad-spectrum antiviral action of IFN-γ may have a correlation with T cell immunity to viral infections. A high level of CD8 T cell immunity and IFN-γ production was reported to be correlated with protection against RSV [42,43]. In previous study, cytotoxic CD8 T lymphocytes and IFN-γ were reported to have dual effects: an effector function for virus control, and immunopathology after infection with RSV [44]. A recent study demonstrated that the ginsenosides from *Panax ginseng* C.A. Meyer reduced IL-4 production but increased IFN-γ production in an ovalbumin-induced murine asthma model [45]. Thus, IFN-γ producing cells in the RGE-treated mice might have contributed to improving the clinical outcomes such as preventing weight loss and enhancing lung viral clearance. Ginseng inhibited the inflammatory responses in lipopolysaccharide-induced macrophage activation *in vitro* and *in vivo* animal models [46]. Oral administration of RGE resulted in an increased survival rates and lowering lung viral loads of mice upon infection with 2009 pandemic H1N1 virus [47]. It is a highly significant finding in this study that the treatment of RGE increased the population of dendritic cells and enhanced the production of antiviral cytokine IFN-γ in BAL fluids upon RSV infection. Dendritic cells are unique professional antigen-presenting cells capable of stimulating naïve T cells in primary immune response, and are more potent than monocyte/macrophages or B cells [48]. Flow cytometry analysis of BAL cells from mice upon RSV infection showed that RGE might be enhancing the cell numbers of CD8^+^ T lymphocytes, early response related-CD11c^+^CD11b^−^ dendritic cells, and late response-dominant related-CD11c^+^CD11b^+^ dendritic cells [49,50]. Furthermore, detailed analysis of BAL cells revealed that RGE treatment enhanced the production of IFN-γ secreting RSV specific-CD4 T cells as well as CD8 T cells. Taken together, RGE-mediated increases in CD8^+^ T cells and CD11c^+^ dendritic cells can be a mechanism contributing to desirable clinical outcomes of diminishing or preventing mouse body weight loss upon infection with RSV even though their molecular mechanisms still remain to be determined. Since ginsenosides are active components of ginseng, it would be informative to test the biological activities of major ginsenosides using the mouse model of RSV infection in the future.

## 5. Conclusions

In summary, this study suggests multiple mechanisms through which ginseng might provide protective efficacy against RSV. RGE inhibited RSV-induced cell death, RSV replication, and the production of pro-inflammatory cytokines *in vitro*. In addition, RGE treatment in mice upon RSV infection resulted in diminishing mouse body weight loss, lowering lung viral loads, and enhancing antiviral IFN-γ production as well as increasing CD8^+^ T cells and CD11c^+^ dendritic cells.
